# A fast and sensitive size-exclusion chromatography method for plasma extracellular vesicle proteomic analysis

**DOI:** 10.1002/pmic.202400025

**Published:** 2024-06-19

**Authors:** Ivo Díaz Ludovico, Samantha M. Powell, Gina Many, Lisa Bramer, Soumyadeep Sarkar, Kelly Stratton, Tao Liu, Tujin Shi, Wei-Jun Qian, Kristin E. Burnum-Johnson, John T. Melchior, Ernesto S. Nakayasu

**Affiliations:** 1 Biological Sciences Division, Pacific Northwest National Laboratory, Richland, Washington, USA; 2 Environmental and Molecular Science Division, Pacific Northwest National Laboratory, Richland, Washington, USA

**Keywords:** biomarker, extracellular vesicles, plasma, proteomics, size-exclusion chromatography

## Abstract

Extracellular vesicles (EVs) carry diverse biomolecules derived from their parental cells, making their components excellent biomarker candidates. However, purifying EVs is a major hurdle in biomarker discovery since current methods require large amounts of samples, are time-consuming and typically have poor reproducibility. Here we describe a simple, fast, and sensitive EV fractionation method using size exclusion chromatography (SEC) on a fast protein liquid chromatography (FPLC) system. Our method uses a Superose 6 Increase 5/150, which has a bed volume of 2.9 mL. The FPLC system and small column size enable reproducible separation of only 50 μL of human plasma in 15 min. To demonstrate the utility of our method, we used longitudinal samples from a group of individuals who underwent intense exercise. A total of 838 proteins were identified, of which, 261 were previously characterized as EV proteins, including classical markers, such as cluster of differentiation (CD)9 and CD81. Quantitative analysis showed low technical variability with correlation coefficients greater than 0.9 between replicates. The analysis captured differences in relevant EV proteins involved in response to physical activity. Our method enables fast and sensitive fractionation of plasma EVs with low variability, which will facilitate biomarker studies in large clinical cohorts.

## 1 | INTRODUCTION

Extracellular vesicles (EVs) are membrane-bound particles released by cells and play a role in tissue crosstalk [1]. EVs are classified based on their biogenesis and size. Exosomes are EV derived from multivesicular bodies ranging from 20 to 300 nm. Ectosomes, however, encompass a wide variety of EVs formed by directing budding from the plasma membrane, such as microvesicles (500–1000 nm) and apoptotic bodies (50–5000 nm) [1, 2]. EVs are composed of metabolites, nucleic acids, soluble and membrane proteins, as well as diverse types of lipids and saccharides [3]. EVs carry molecular signatures from their parental cell, presenting immense potential for biomarker development [3]. However, the purification step is a major hindrance to perform large-scale biomarker studies targeting EVs.

Current EV purification methods require large sample amounts, which is an impairment for most clinical studies. In addition, these methods have low performance, requiring methodological improvements. First, EVs share similar physicochemical properties with other biofluid components such as albumin and lipoproteins making EV purification difficult as they are minor components of biofluids, displaying one order of magnitude lower abundance than plasma lipoproteins [4]. Sequential purification steps can increase preparation purity, but result in high sample loss (≥99% after two purification steps) and are very time-consuming [5]. To better understand this issue, we recently performed a proteomics meta-analysis of EV datasets obtained with different purification methods, leading to the identification of 1717 proteins with a high probability to be EV proteins [6]. With improved characterization of plasma EV proteins and the ability to distinguish them from contaminants, the purity of the samples became less important for quantitative proteomics analysis, and therefore our efforts were focused on improving EV recovery. By analyzing different methods, it was found that size-exclusion chromatography (SEC) results in the highest EV recovery, averaging 35% of the proteome abundance [6].

**FIGURE 1:**
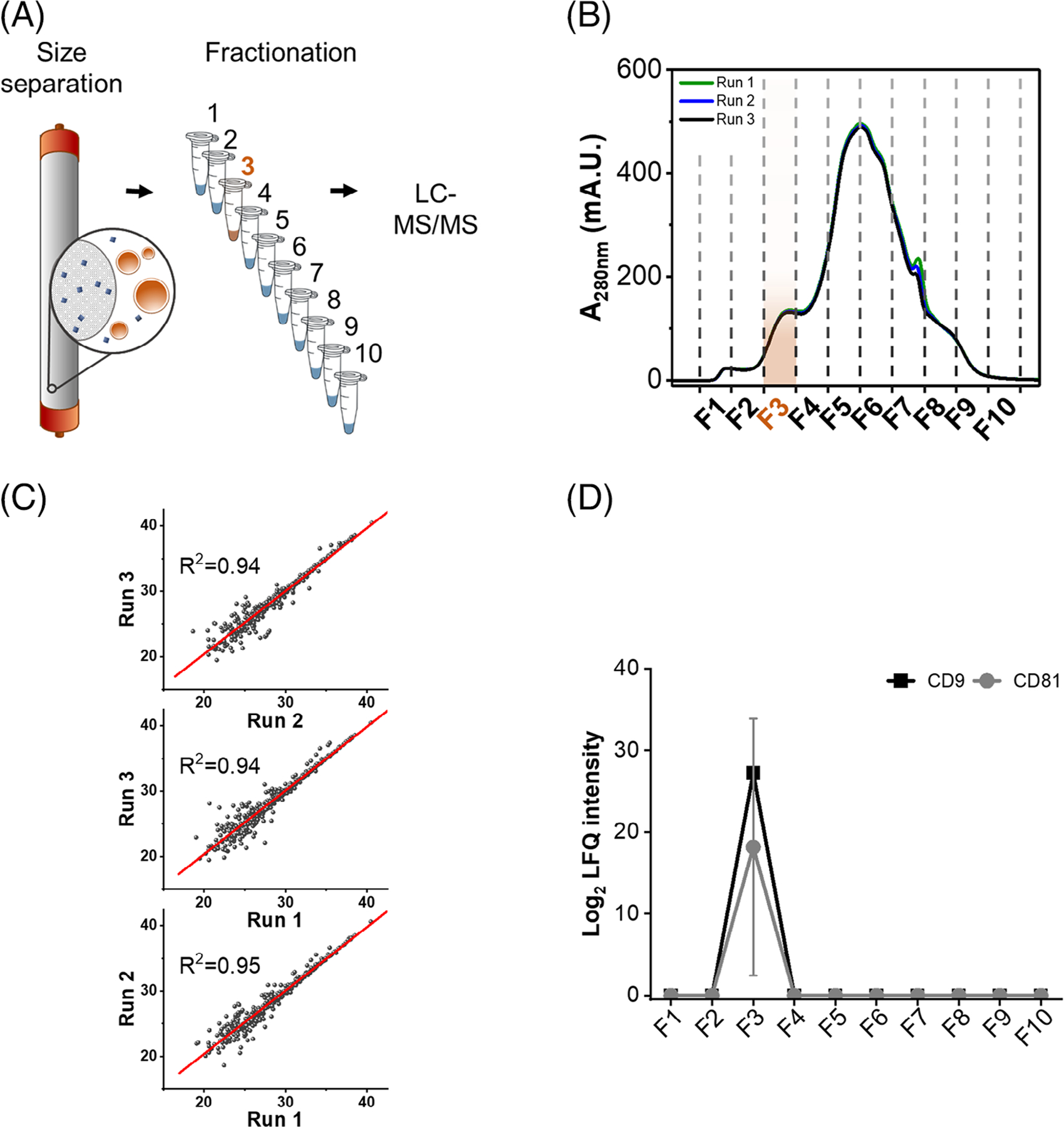
Human plasma extracellular vesicle (EV) fractionation by size exclusion chromatography. (A) Scheme of human plasma fractionation by size exclusion chromatography in a Superose 6 GE 5/150 column. (B) Chromatograms resulting from three consecutive runs. Fraction collection is delimited by dashed lines and the fraction containing EV is highlighted in orange. (C) Scatter plots of Log_2_ intensity of identified proteins comparing the three different runs. Linear regression curves (red) and coefficient of determination (R^2^) are also shown for each plot. (D) Elution profiles of the EV markers cluster of differentiation (CD)81 and CD9 are shown as average and standard deviation from three consecutive runs. LC-MS/MS, liquid chromatography-tandem mass spectrometry; LFQ, label-free quantification; mA.U., absorbance units (×10^−3^).

Most of current SEC-based EV purification methods require approximately 1 mL of human plasma [7]. Recently, Lattmann et al. performed EV proteomics with 200 μL of plasma [8], representing a major development in the field. However, for large research consortia on which a small sample is collected for multiple analyses, this volume requirement is still prohibitive. Here, we aimed to further develop the technique to require smaller sample volumes. We based our method on the principle of reducing sample preparation volumes to reduce sample losses [9, 10]. We used a prepackaged SEC column (Superose 6 GE increase 5/150, Cytiva), which has a bed volume of only 2.9 mL compared to 24 mL of the most common 10 mm × 30 cm columns. We performed the SEC fractionation on a fast protein liquid chromatography system (FPLC; ÄKTApurifier^™^, Cytiva) to improve the speed and reproducibility. To determine the feasibility of this down-scaled approach, we injected 50 μL of human plasma (BioIVT) into the column (Figure 1A). The elution was performed with an isocratic gradient of 10 mM Tris-HCl, pH 7.5 with 150 mM NaCl, 1 mM ethylenedi-aminetetraacetic acid, 0.02% sodium azide at 0.3 mL/min, resulting in remarkably similar elution profiles across three runs (Figure 1B). Samples were collected during elution into 10 fractions of 0.3 mL each. SEC fractionation was then assessed by proteomic analysis.

For protein digestion, SEC fractions were submitted to a 30 min incubation in the dark with 1% sodium dodecylsulfate, 2 mM tris(2-carboxyethyl)phosphine, and 1 mM iodoacetamide. Each sample was then precipitated with 80% acetone overnight at −20°C and recovered by centrifugation at 4°C at 20,000 × *g* for 30 min. Pellets were washed with cold acetone (−20°C) and recovered by centrifuging for 10 min at 20,000 × *g* and 4°C. They were dried at room temperature for 30 min, dissolved in 10% trifluoroethanol in 100 mM NH_4_HCO_3_, and digested with 1:100 (enzyme:protein) endoproteinase LysC (Promega) for 2 h at 37°C with 800 rpm shaking and followed by 1:50 (enzyme:protein) trypsin (Promega) for 16 h at 37°C with 800 rpm shaking.

LC-MS-MS analysis of the peptides was performed on a Waters NanoAquity UPLC system (Waters Corporation) with a C18 column (70 cm × 75 μm i.d., custom packed Jupiter 3 μm particle size, 300Å pore size, Phenomenex) coupled with a Q-Exactive mass spectrometer (Thermo Scientific). Peptide separation was carried out with a gradient of water (solvent A) and acetonitrile (solvent B) both containing 0.1% formic acid (1%–8% B in 2 min, 8%–12% B in 18 min, 12%–30% B in 55 min, 30%–45% B in 22 min, 45%–95% B in 3 min, hold for 5 min in 95% B and 99%–1% B in 10 min). Eluting peptides were directly analyzed by nanoelectrospray ionization with a scan range of 300–1800 m/z and 70,000 resolution at 400 m/z. The top 12 most intense parent ions were submitted to data-dependent acquisition (DDA) using high-energy collision-induced dissociation fragmentation (2.0 m/z isolation width; 30% normalized collision energy; 17,500 resolution at 400 m/z) with a 50,000 intensity threshold before being dynamically excluded for 30 s. Subsequent data processing with MSFragger in FragPipe v19.0 [11] against the human reference proteome (Uniprot Knowledgebase, downloaded January 2023), considered fully tryptic peptides, cysteine carbamidomethylation (fixed modification), and methionine oxidation and protein N-terminal acetylation (variable modifications). The analysis led to the identification of 539 proteins with correlation between replicates ≥0.94 (Figure 1C). When compared to the EV proteins from our previous meta-analysis study [6], we detected a total of 95 EV proteins across the proteome (Table S1), and they were significantly enriched with EV proteins (Table S2). We also assessed the distribution of EV across the fractions based on the classical markers, the cluster of differentiation (CD)9 and CD81. These markers were only observed in fraction 3 (Figure 1D), suggesting that EV eluted exclusively in that fraction.

**FIGURE 2:**
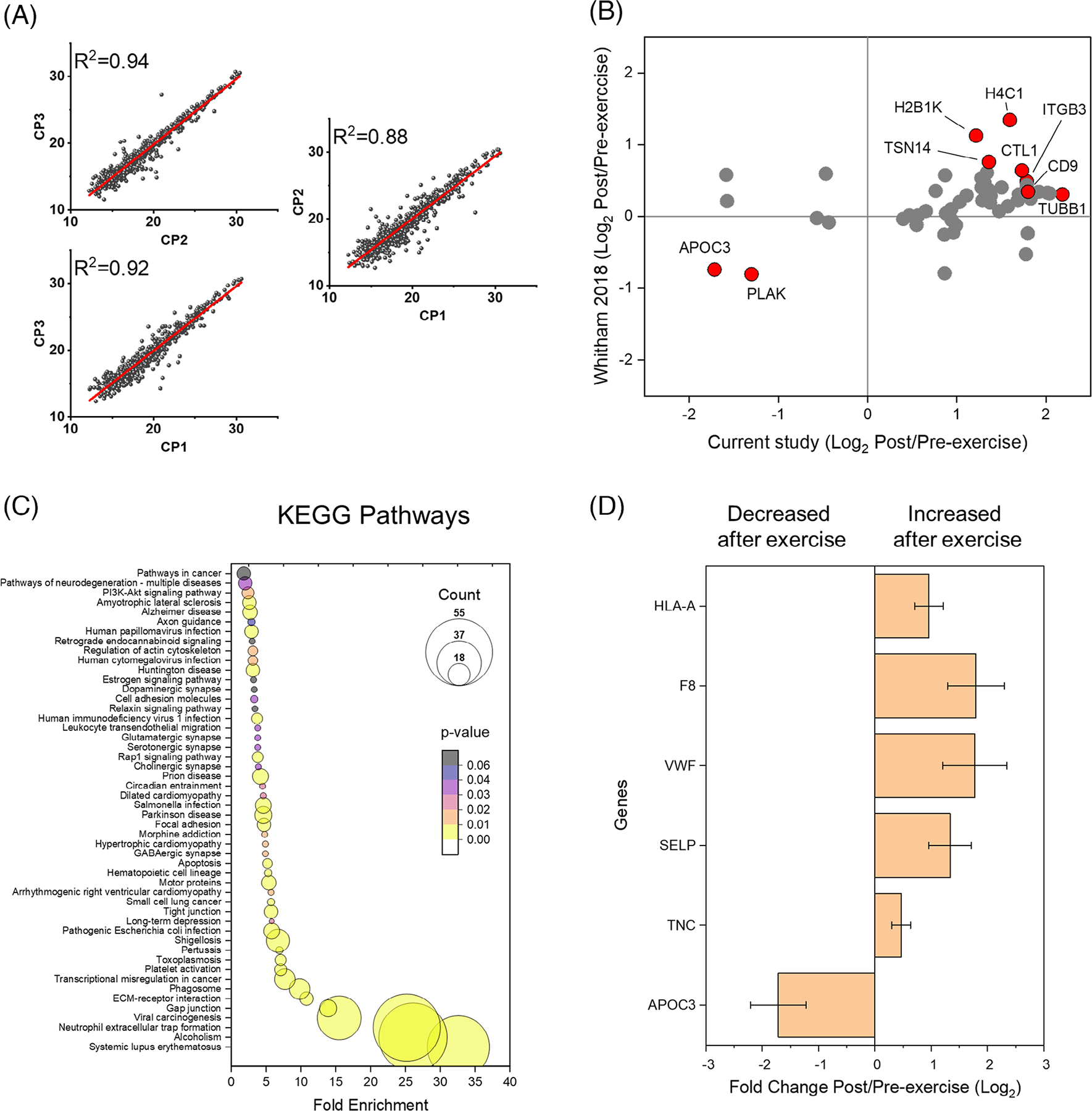
Analysis of plasma extracellular vesicles from a cohort of firefighters that underwent an intense exercise bout. (A) Scatter plots of averaged Log_2_ intensity of identified proteins comparing three commercial plasma (CP) runs, analyzed at the beginning, middle and end of the experiment, linear regression curves (red) and coefficient of determination (R^2^) are also shown for each plot. (B) Cross-validation of differentially abundant proteins post-exercise found in common with Whitham et al. 2018 [1]. Examples of proteins with similar regulation between both studies are highlighted in red. (C) Functional-enrichment analysis of proteins regulated post-exercise. (D) Examples of regulated proteins that are relevant to exercise physiology: immunoregulatory HLA class I histocompatibility antigen, A (HLA-A); coagulation factor VIII (F8); von Willebran factor (VWF); P-selectin (SELP); tenascin C (TNC); and apolipoprotein C-III (APOC3).

We further tested our pipeline by examining the EV response in 50 μL of plasma from nine firefighters immediately before and after an acute (45-min) bout of exercise obtained as described before [12]. Acute exercise has been previously shown to mobilize EV, largely from the skeletal muscle, into circulation [13]; and their protein cargo is known to play an important role in exercise physiology [14]. In the chromatograms, we observed a net increase in the absorbance of fractions corresponding to EV elution in six out of nine individuals post-exercise (Figure S1), supporting previous reports of EV mobilization following an acute bout of strenuous exercise [1]. The EV-enriched fractions were prepared for LC-MS/MS analysis by acetone precipitation followed by S-trap digestion to further minimize sample loss [15] and were analyzed by data independent acquisition (DIA) on a NanoAcquity UPLC system connected to Q-Exactive HFX mass spectrometer (Thermo Scientific). The chromatographic conditions were identical as described above. We built a peptide library by using pooled aliquots of each sample that were submitted to micro-fractionation [16] and DDA analysis as described above. Individual samples were analyzed by DIA with a 400–900 m/z range and 10 m/z increment windows (30% normalized collision energy; 70,000 resolution at 400 m/z). Both DDA and DIA data were analyzed together in FragPipe with similar parameters described above, and quantitative information was extracted with DIA-NN [17]. A total of 838 proteins were identified, of which 261 proteins (32%) are known to be EV proteins based on our previous meta-analysis (Table S3) [6]. To test the variability of our platform, we randomized, prepared, and analyzed in parallel a commercial human plasma sample, used as a measure of quality control. The quality control sample had a correlation average of 0.91 (Figure 2A), indicating low variability of the platform.

Given the repeatability of our platform, we next examined its utility to provide biological insight into the acute exercise response via statistical and functional-enrichment analyses. In response to acute exercise, we observed 10 downregulated and 55 upregulated proteins in EV-fractions (*p* < 0.05) (Table S4). We next cross-validated biomarker candidates by re-analyzing the plasma EV proteomic response to acute aerobic exercise from a separate cohort of subjects [1]. Out of the regulated proteins, 57 EV proteins were also detected in the data from the previous work, and 43 proteins displayed a similar temporal response to acute exercise despite differences in exercise regimen. Here we observed an increased abundance of the EV marker CD9 and a decreased abundance of apolipoprotein C-III (APOC3) (Figure 2B). APOC3 is a lipoprotein lipase inhibitor [18], and its downregulation thus permits lipoprotein lipase-mediated fatty acid release to meet the energetic demands of the exercise.

Subsequent functional-enrichment analysis [19] of the 65 exercise-responsive EV proteins in our data set revelated enrichment of 50 pathways following acute exercise (Figure 2C), highlighting candidate biological functions for exercise-related EVs. Upregulated pathways included pathways involved in acute inflammatory responses, such as neutrophil activation and coagulation. The acute inflammatory and pro-thrombotic response to strenuous exercise is well documented [20–22]. In agreement, we detected an increased abundance of coagulation EV proteins, including coagulation factor VIII (F8), P-selectin (SELP) and von Willebran factor (VWF), immunoregulatory HLA class I histocompatibility antigen, A (HLA-A), and tenascin C (TNC), a marker of muscle damage [23], following acute exercise (Figure 2D). These results indicate that our platform can also provide insights into the biological roles of EV.

In this work, we developed a SEC platform for EV fractionation that is fast (15 min), robust, has low variability, and dramatically reduces the required sample volume to 50 μL. The application of this platform to a cohort of firefighters that underwent strenuous physical training showed key proteins mobilized in EV in response to acute exercise. We were also able to cross-validate biomarker candidates with data from a previous study, demonstrating the potential applicability of this method for clinical studies. We confirmed the method’s robustness by analyzing samples from multiple individuals. Although this platform has not yet been tested for large-scale applications, as the method is currently designed, it could be fully automated, allowing for easy application of the method to large-scale studies.

## Supplementary Material

Supplementary Material

Supplemental Figure 1

## Data Availability

Mass spectrometry data are publicly available in MassIVE repository, a member of the ProteomeXchange Consortium, under the accession number MSV000093427.

## Significance Statement

Biomarker discovery through EV analysis is a promising area for the diagnosis and prognosis of numerous diseases, yet the currently available methodologies create a bottleneck, slowing discovery. EVs share physicochemical properties with other particles and molecules in human biofluids, often making the enrichment process time and sample consuming, and poorly reproducible. In our previous work, we performed a careful characterization of the plasma EV composition (https://doi.org/10.1038/s41597-023-02748-1), which allows us now to focus our efforts to develop proteomics methods to address the sensitivity, reproducibility and time issues, in despite of the sample purity. Here we present a method that reduces the required plasma amount by up to 40 times when compared to current protocols, and processing time to only 15 min. Moreover, we have demonstrated that the method detects changes across physiologically relevant processes, which represent biomarker candidates. The low variability, low sample volume requirements, and reduced processing times open opportunities to apply this method in large clinical studies for biomarker development.

## Abbreviations:

CD81: cluster of differentiation 81
CD9: cluster of differentiation 9
DDA: data-dependent acquisition
DIA: data-independent acquisition
EV: extracellular vesicles
FPLC: fast protein liquid chromatography
SEC: size-exclusion chromatography
