# Supplementary material for: A fast and sensitive size-exclusion chromatography method for plasma extracellular vesicle proteomic analysis
Source: Proteomics. Author manuscript; Available in PMC 2025 Aug 1. (PMC11610398; doi:10.1002/pmic.202400025)
Supplement: Supplemental Figure 1 [file NIHMS2034061-supplement-Supplemental_Figure_1.docx]

**Supplementary material**

A fast and sensitive size-exclusion chromatography method for plasma extracellular vesicle proteomic analysis

Ivo Díaz Ludovico^1^, Samantha M. Powell^1^, Gina Many^1^, Lisa Bramer^1^, Soumyadeep Sarkar^1^, Kelly Stratton^1^, Tao Liu^1^, Tujin Shi^1^, Wei-Jun Qian^1^, Kristin E Burnum-Johnson^2^, John T. Melchior^1^, Ernesto S. Nakayasu^1*^

^1^Biological Sciences Division, Pacific Northwest National Laboratory, Richland, WA, USA.

^2^Environmental and Molecular Science Division, Pacific Northwest National Laboratory, Richland, WA, USA.

**
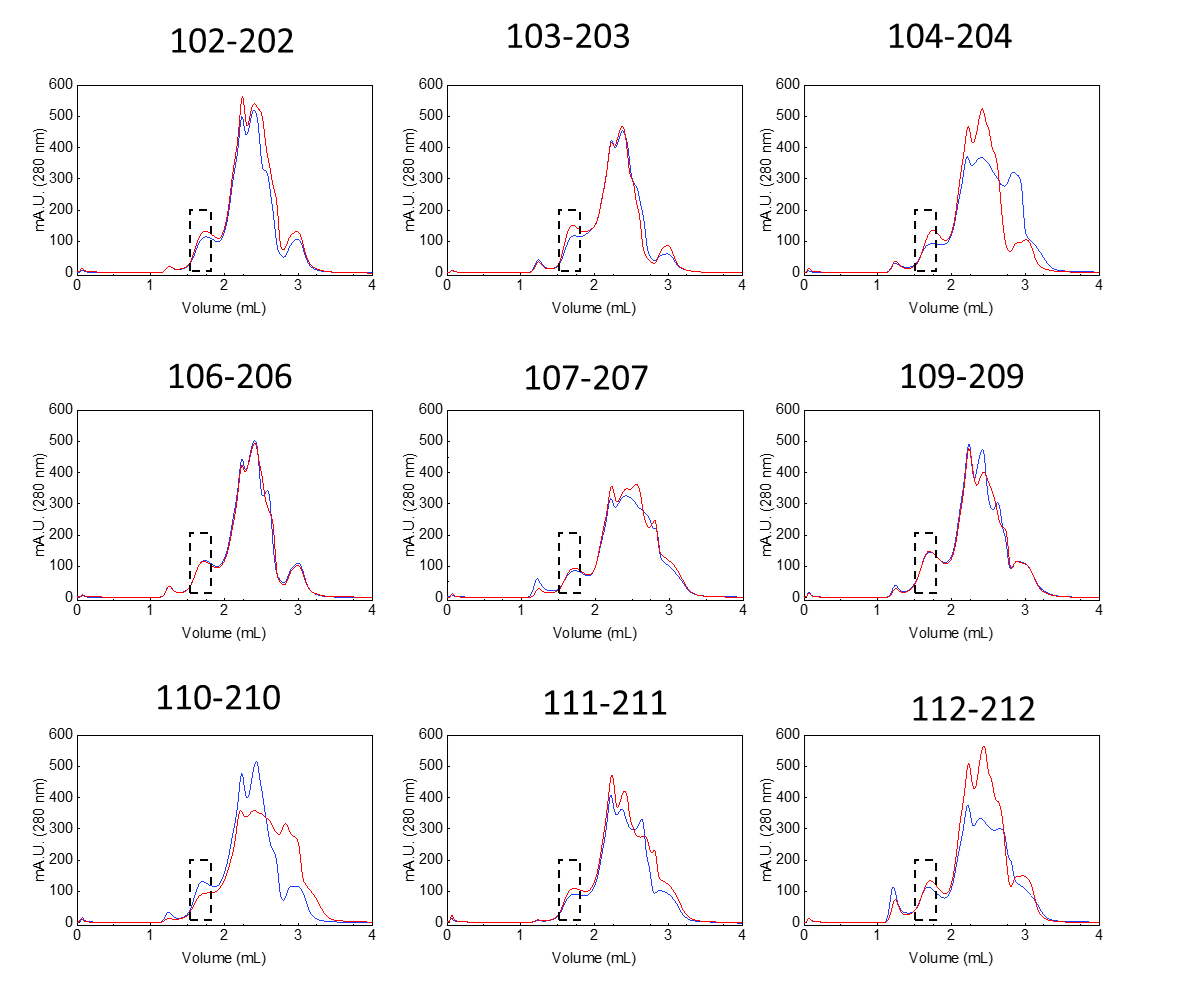
**

**Supplementary Figure 1 – Chromatographic profile of plasma from firefighter volunteers fractionated by size exclusion chromatography.** 50 µL of human plasma pre- (BLUE) and post- (RED) exercise were injected into a Superose 6 increase 5/150 and separated in an isocratic gradient at a flow rate of 0.3 mL/min. Fraction number 3 (dashed box) of pre- and post- exercise of each individual was submitted to proteomics analysis.
